# Potential Risks of Severe Infection Following the Exploratory Laparoscopy for Advanced Ovarian Cancer: A Case Report and a Literature Review

**DOI:** 10.7759/cureus.65415

**Published:** 2024-07-26

**Authors:** Yuka Fukunishi, Shintaro Yanazume, Chikako Nagata, Mika Mizuno, Shinichi Togami, Hiroaki Kobayashi

**Affiliations:** 1 Obstetrics and Gynecology, Kagoshima University Hospital, Kagoshima, JPN

**Keywords:** abscess, ovarian cancer, infection, exploratory laparoscopy, advanced

## Abstract

Although exploratory laparoscopy in patients with advanced ovarian cancer is a diagnostic tool for determining treatment strategy, its safety has not been completely investigated. We report a case involving a severe abdominal abscess following an exploratory laparoscopy. A 65-year-old woman with advanced ovarian cancer developed a large abdominal abscess following exploratory laparoscopy and neoadjuvant chemotherapy. Emergent laparotomy was performed; while massive bowel adhesion surrounding the abscess did not allow for genital organ resection, an incision in the left port area was made to drain the abscess. The patient’s chemotherapy was delayed because she experienced sub-ileus, postoperatively. Only a limited number of studies have been conducted on the safety of these techniques. This intense infection case emphasizes the need for further investigations into the safety of exploratory laparoscopy in patients with progressive diseases under heterogeneous conditions in real-world settings.

## Introduction

Exploratory laparoscopy is now standard for decisions regarding treatment strategy in cases of advanced ovarian cancer complicated with peritoneal dissemination [1]. This procedure is considered in patients where it is not possible to determine the extent of the tumor from imaging and to ascertain whether optimal surgery is possible. The procedure is also performed to ensure that chemotherapy-naïve tissue is available for genetic testing in patients where neoadjuvant chemotherapy (NAC) has been recommended. This helps to guide treatment decisions and improve patient outcomes.

Despite exploratory laparoscopy being a commonly used diagnostic tool for advanced ovarian cancer, clinical investigations into its safety have not been entirely investigated, and the available information is based on examinations conducted prior to the 2000s for the treatment of early ovarian cancer [2,3]. Recent research has centered on the significance of staging laparoscopy in predicting the attainment of optimal surgical outcomes, as demonstrated by Fagotti et al.'s development of the predictive Index score. Eligible patients for the latter study were those who were clearly in a suitable condition for laparoscopy [4-6]. Limited evidence exists regarding the safety of laparoscopy for patients with progressive diseases under heterogeneous conditions such as undernutrition and immunocompromise in real-world settings.

We hereby present a case report of a patient who developed a large abdominal abscess following exploratory laparoscopy for advanced ovarian cancer.

## Case presentation

The patient, a 65-year-old nulliparous woman, 162 cm in height, and 57 kg in weight, was urgently referred to our hospital for invasive treatment of a severe infection during NAC following exploratory laparoscopy for advanced ovarian cancer. She had no diabetes or immunosuppressive conditions. Full informed consent was obtained from the patient. This report has been approved by a suitably constituted Ethics Committee of the Institutional Review Board of Kagoshima University Graduate School of Medical Sciences (approval #230340) within which the work was undertaken and it conforms to the provisions of the Declaration of Helsinki in 1995 (as revised in Brazil 2013).

Three months prior to hospital admission, the patient had visited a clinic with complaints of abdominal distension and loss of appetite. She was promptly transferred to a nearby general hospital, where a computed tomography (CT) scan revealed the peritoneum dissemination throughout the entire abdominal cavity, accompanied by massive ascites. The patient was unable to be transported to a more advanced medical facility due to her poor general condition, which was classified as performance status (PS) 3. Although ovarian or peritoneal cancer was suspected, CT was non-contrasted because of high serum creatinine levels and accurate assessment was difficult. Laparoscopic exploration was scheduled to provide a definitive diagnosis and to assess the feasibility of optimal surgical intervention. Surgery was performed jointly by a surgeon and an obstetrician-gynecologist, without any apparent complications during the procedure. Adnexal mass was 5cm and invaded surrounded organs, ometal cake with 8cm diameter was diffusely present. The postoperative course was uneventful. A diagnosis of high-grade serous carcinoma of ovarian cancer stage ⅢC was confirmed, and NAC with paclitaxel and carboplatin (TC) was initiated on postoperative day 14. Due to poor PS, the patient was retained in admissions. On day 10 of the second course, she experienced febrile neutropenia with a fever >39°C, and broad-spectrum antibiotics with cefepime and granulocyte colony-stimulating factor (G-CSF) were administered. Serum CA125 levels were reduced from 448 U/mL before chemotherapy to 60.4 U/mL following chemotherapy, while infection treatment was maintained.

**Figure 1:**
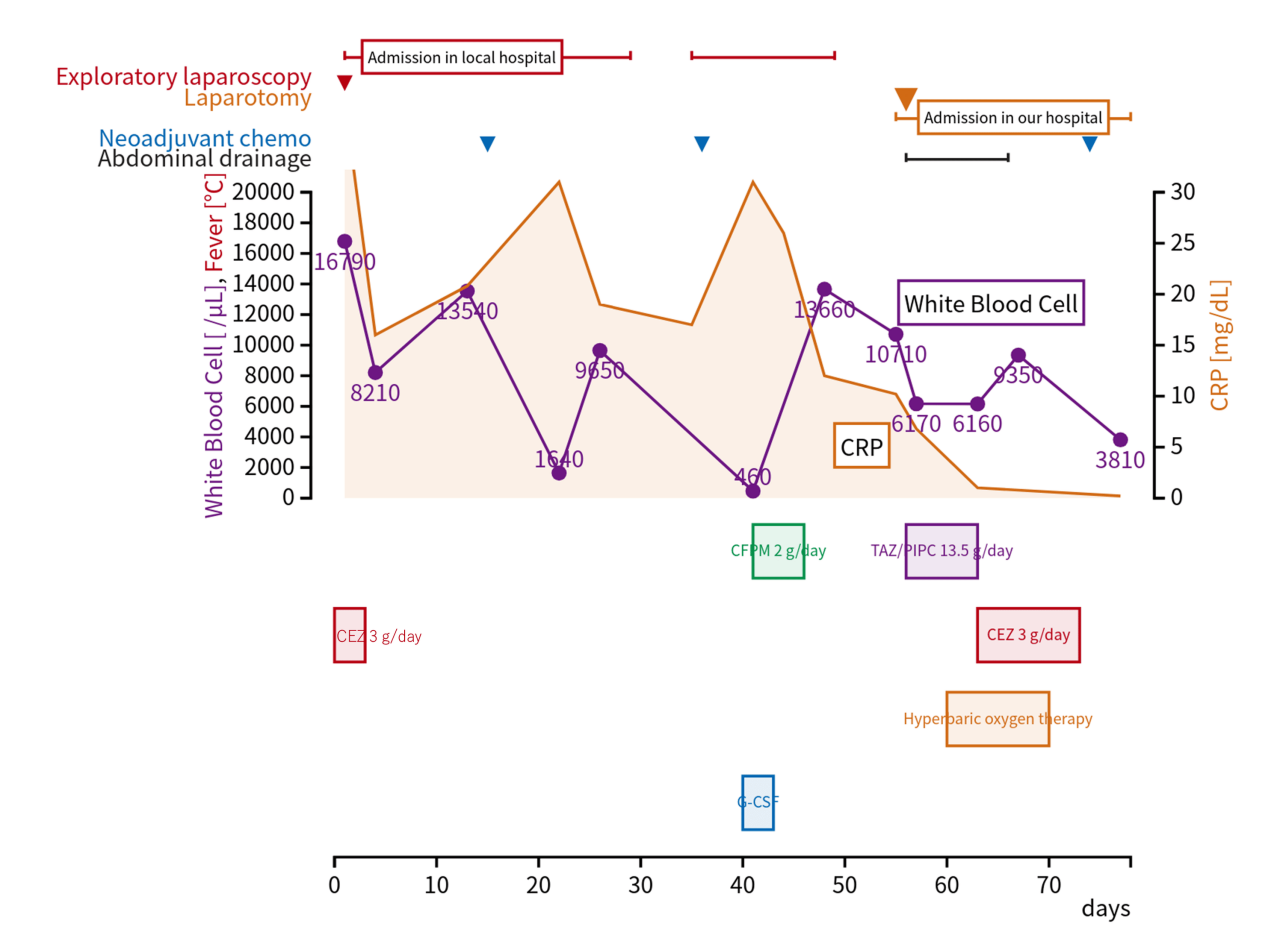
Clinical course of the patient with abdominal abscess CRP: C-reactive protein; G-CSF: granulocyte colony-stimulating factor; CFPM: cefepime; TAZ: tazobactam; CEZ: ceftazidime; PIPC: piperacillin

The patient was referred to our medical center for management of persistent abdominal discharge and infection. The clinical course of the patient is shown in Figure 1.

**Figure 2:**
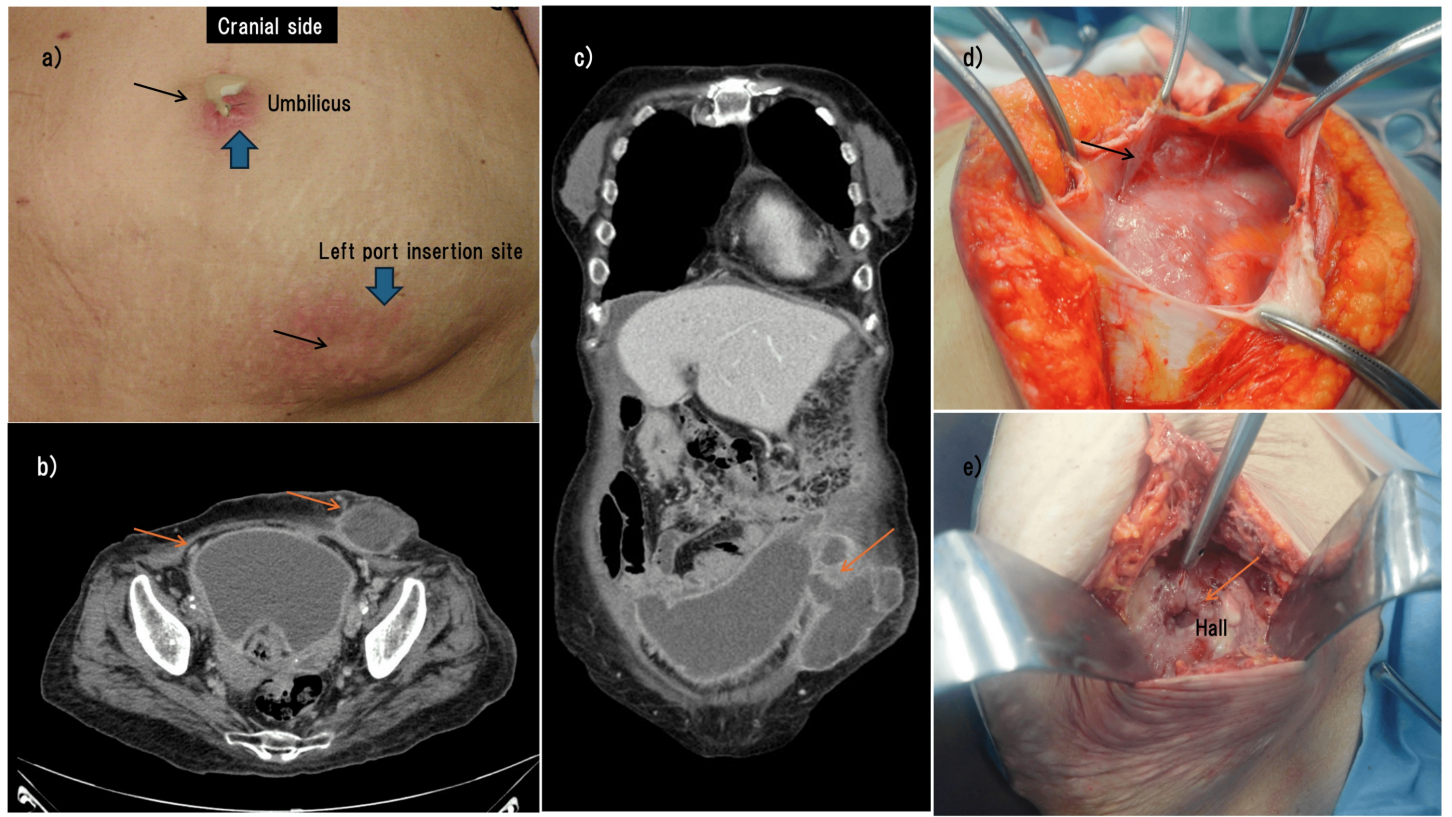
Clinical findings of the patient with abdominal abscess (a) Abscess was flowing from the umbilicus (arrow) or the laparoscopic port insertion site (arrow) in the lower abdomen; (b) The CT scan revealed an irregularly shaped subcutaneous abscess extending pelvic cavity (arrows), measuring 15×20×12 centimeters; (c) The subcutaneous abscess communicates with the pelvic abscess (arrow); (d) Massive bowel adhesion surrounding the abscess (arrow); (e) Opening the subcutaneous abscess and a hall reached the pelvic cavity was shown (arrow).

During a physical examination, her abdomen was distended complicated with slight tenderness, and a yellowish discharge flowed from the umbilicus or the laparoscopic port insertion sites in the lower abdomen (Figure 2a). Fever, serum Hb, WBC, and C-reactive protein levels were 37.5°C, 9.7 g/dL, 10 710/mL, and 10.24 g/dL, respectively. The CT scan revealed a substantial, irregularly shaped abscess that extended from the pelvic region to the subcutaneous tissue beneath the left port insertion point, measuring 15×20×12cm (Figures 2b-2c). Emergent laparotomy was performed; however, massive bowel adhesion surrounding the abscess did not allow for genital organ resection (Figure 2d). We ceased performing hysterectomy and salpingo-oophorectomy and instead made an incision in the left port area to drain the abscess (Figure 2e).

On the third day of her postoperative course, the patient experienced sub-ileus, and hyperbaric oxygenation was performed. The postoperative exudate continued to drain for a week, and the chemotherapy schedule that was originally planned was delayed and performed on postoperative day 17. An ascitic fluid culture, which was obtained from a drain on day 7 following an exploratory laparotomy, showed significant levels of *Staphylococcus aureus* (methicillin-sensitive *S. aureus*, MSSA), and the same bacterium was identified in samples obtained from the abscess drainage.

## Discussion

Exploratory laparoscopy has been established as the standard approach for identifying patients who are suitable candidates for complete cytoreduction surgery in advanced ovarian cancer. This case indicates that advanced, progressive conditions may not always be investigated safely through exploratory laparoscopy. Even if a patient does not have diabetes or immunosuppression, infection can surface and become serious during postoperative chemotherapy.

Previous randomized controlled studies (RCTs) have shown that NAC followed by interval debulking surgery in patients with stage ⅢC or Ⅳ ovarian cancer is not inferior to primary debulking surgery (PDS) in terms of overall survival, and surgical invasiveness. Moreover, patients who received NAC experienced a lower frequency of abdominal organ resection and distant metastases resection, as well as a reduced incidence of adverse events related to treatment. Specifically, patients who underwent NAC had less blood loss, required fewer albumin transfusions, and experienced fewer grade Ⅲ or Ⅳ adverse events after surgery. Overall, these findings suggest that NAC followed by interval debulking surgery may be a more effective and less invasive treatment option for patients with stage ⅢC or Ⅳ ovarian cancer [6-8]. However, the completion rates and optimal results of these trials have not been satisfactory because of the rate of diagnostic laparoscopy before treatment. The use of exploratory laparoscopy in assessing patients with high tumor load via a predictive index scoring system has demonstrated high optimal surgery rates, highlighting its importance [6].

The safety of exploratory laparoscopy has not been completely investigated. This is evidenced by a review of the literature as shown in Table 1 [4,5,9-23].

**Table 1:** The review of the feasibility findings caused by exploratory laparoscopy in advanced ovarian cancer RCT: randomized controlled trial; PCI: Peritoneal Cancer Index

Authors	Years	Cases	Design	Main findings	Safety comments
Vergote et al. [9]	1998	77	Observational study	Neoadjuvant chemotherapy is associated with higher crude survival compared to primary debulking surgery.	Six patients developed subcutaneous metastasis at the site of one of the trocars.
Fagotti et al. [4]	2005	64	Observational study	Laparoscopy can be considered superimposable to standard laparotomy in identifying not optimally resectable cases.	Nil
Deffieux et al. [10]	2006	15	Observational study	Laparoscopy may obviate the need for unnecessary laparotomy in many cases.	No port-site metastasis occurred.
Angioli et al. [11]	2006	87	Observational study	Diagnostic open laparoscopy is a valid diagnostic tool, reducing primary cytoreductive surgery rates while achieving higher optimal debulking rates at primary surgery.	In two cases (6%), a trocar metastasis was found, both of them at the level of the ancillary trocar site insertion.
Fagotti et al. [12]	2008	113	Observational study	The proposed laparoscopic model appears a reliable and flexible tool to predict optimal cytoreduction.	No intraoperative complications were registered.
Brun et al. [13]	2008	55	Observational study	A simplified laparoscopy-based score, incorporating four parameters, is as accurate as the Fagotti score to predict resectability.	Nil
Chéreau et al. [5]	2010	61	Observational study	Alternative peritoneal carcinomatosis scoring systems provide additional information for predicting complete resectability, complications, and survival.	Nil
Varnoux et al. [14]	2013	28	Observational study	Hand-assisted laparoscopy may perform better than laparoscopy alone for predicting the resectability of peritoneal carcinomatosis by increasing the number of sites evaluated.	Nil
Petrillo et al. [15]	2015	234	Observational study	The updated staging laparoscopy accurately predicts incomplete cytoreduction, reducing unnecessary laparotomy explorations.	Nil
Rutten et al. [21]	2016	201	RCT	Diagnostic laparoscopy can reduce the number of futile laparotomies, considering primary treatment decisions.	Only one complication was related to laparoscopy: a wound infection that required antibiotics. Port-site metastases after laparoscopy were reported in three patients.
Dessapt et al. [16]	2016	123	Observational study	Age >60 years, diaphragmatic carcinomatosis, and a PCI>10 are independently associated with incomplete cytoreductive surgery.	Nil
Rosetti et al. [14]	2016	52	Observational study	Laparoendoscopic single-site surgery is a feasible, safe, and effective minimally invasive procedure for assessing peritoneal carcinomatosis resectability.	No grade 3 or 4 perioperative complications were observed.
Eoh et al. [17]	2017	38	Observational study	The consecutive steps of imaging, frailty assessment, and diagnostic laparoscopy might facilitate rapid assessments of peritoneal disease extent and resectability.	Nil
Vizzielli et al. [18]	2016	555	Observational study	The laparoscopic score (i.e.: predictive index value) may accurately predict a patient's postoperative outcome. Early identification of high-risk patients could help the surgeon to adopt tailored strategies on an individual basis.	Nil
Hansen et al. [19]	2018	226	Observational study	Laparoscopic assessment of tumor burden is a feasible tool for predicting complete cytoreduction (residual disease of 0), with concordance varying by anatomical location.	Nil
Odajima et al. [23]	2021	23	Observational study	Laparoscopic tumor biopsy is useful and safe for histological diagnosis, thereby allowing for an early introduction to neoadjuvant chemotherapy.	There was no recurrence at the port site in the laparoscopy group during the follow-up period.
Lee et al. [20]	2023	614	Observational study	Diagnostic laparoscopy reduces suboptimal cytoreduction rates and neoadjuvant chemotherapy implementation, while reducing postoperative morbidity without affecting survival outcomes.	There were no serious laparoscopy-related complications in any patient.

Most articles focus on estimating the optimal surgery rates or accuracy of staging, while only a few reports address the safety of these procedures [9-12,20-23]. Some studies have pointed out the existence of port-site metastases, which are commonly regarded as a critical complication that demands prompt attention. Among the descriptions of complications except for port-site metastases [12,20-23], only a case with a wound infection that required antibiotics was reported [21].

 In our case, we reevaluated the video of the exploratory laparoscopy, and we were unable to identify any damage to the bowel or genitourinary organs. Poor PS is considered as a risk factor for promoting infection. Our case involved a patient with a poor general condition (PS3). The first course of NAC was delayed due to the patient's postoperative condition being unfavorable. Among the published clinical trials, the JCOG0602 study comprised a patient population of 12.8% to 13.8% classified as PS2-3, which is in close agreement with real-world data. In contrast, most other RCTs enrolled patients with a PS ≤2, reflecting a generally favorable clinical condition [6-8]. Our patient’s abscess was deemed to have originated from beneath the skin, as indicated by the CT scan; there has also been a previously documented case of port-site metastasis forming beneath the skin [9]. The port insertion site could have been the root cause of the complications. Low nutrition and low immunity during chemotherapy contributed to infection. However, less invasive endoscopic surgery has the advantage of allowing patients to perform immediately to chemotherapy.

## Conclusions

Postoperative infections are a potential complication in patients who undergo exploratory laparoscopy due to the administration of chemotherapy immediately following surgery. Early detection and prompt treatment are important to prevent disease deterioration. This is a short case report, and there is currently limited detailed information regarding the mechanism of infection following exploratory laparoscopy, so future trials may warranted to confirm the safety and efficacy of exploratory laparoscopy in advanced ovarian cancer.
